# Epigenome Defines Aberrant Brain Laterality in Major Mental Illnesses

**DOI:** 10.3390/brainsci14030261

**Published:** 2024-03-07

**Authors:** Hamid Mostafavi Abdolmaleky, Shabnam Nohesara, Sam Thiagalingam

**Affiliations:** 1 Department of Medicine (Biomedical Genetics), Boston University Chobanian and Avedisian School of Medicine, Boston, MA 02118, USA; snohesar@bu.edu; 2 Department of Surgery, Nutrition/Metabolism Laboratory, BIDMC, Harvard Medical School, Boston, MA 02215, USA; 3 Department of Pathology & Laboratory Medicine, Boston University Chobanian and Avedisian School of Medicine, Boston, MA 02118, USA

**Keywords:** epigenetic, brain, laterality, asymmetry, schizophrenia, bipolar disorder, OCD, ADHD, autism, *TGFB2*

## Abstract

Brain-hemisphere asymmetry/laterality is a well-conserved biological feature of normal brain development. Several lines of evidence, confirmed by the meta-analysis of different studies, support the disruption of brain laterality in mental illnesses such as schizophrenia (SCZ), bipolar disorder (BD), attention-deficit/hyperactivity disorder (ADHD), obsessive compulsive disorder (OCD), and autism. Furthermore, as abnormal brain lateralization in the planum temporale (a critical structure in auditory language processing) has been reported in patients with SCZ, it has been considered a major cause for the onset of auditory verbal hallucinations. Interestingly, the peripheral counterparts of abnormal brain laterality in mental illness, particularly in SCZ, have also been shown in several structures of the human body. For instance, the fingerprints of patients with SCZ exhibit aberrant asymmetry, and while their hair whorl rotation is random, 95% of the general population exhibit a clockwise rotation. In this work, we present a comprehensive literature review of brain laterality disturbances in mental illnesses such as SCZ, BD, ADHD, and OCD, followed by a systematic review of the epigenetic factors that may be involved in the disruption of brain lateralization in mental health disorders. We will conclude with a discussion on whether existing non-pharmacological therapies such as rTMS and ECT may be used to influence the altered functional asymmetry of the right and left hemispheres of the brain, along with their epigenetic and corresponding gene-expression patterns.

## 1. Introduction

The loss or reversal of handedness and the normal structural and functional pattern of brain asymmetry is one of the most consistent brain anomalies in major mental illnesses such as SCZ [1]. In addition to SCZ, patients with BD exhibit noticeable hemispheric differences and/or a reversal of left–right asymmetry in some brain areas compared to control subjects [2]. Patients with major depressive disorder (MDD) have also demonstrated functional and structural asymmetry of the dorsolateral prefrontal cortex [3]. Moreover, one of the features of ADHD, OCD, and autism spectrum disorders (ASD) is a disruption in the lateralized neurodevelopmental skills and changes in structural brain asymmetry which influence vast brain areas along with various corresponding functions [4].

**Figure 1:**
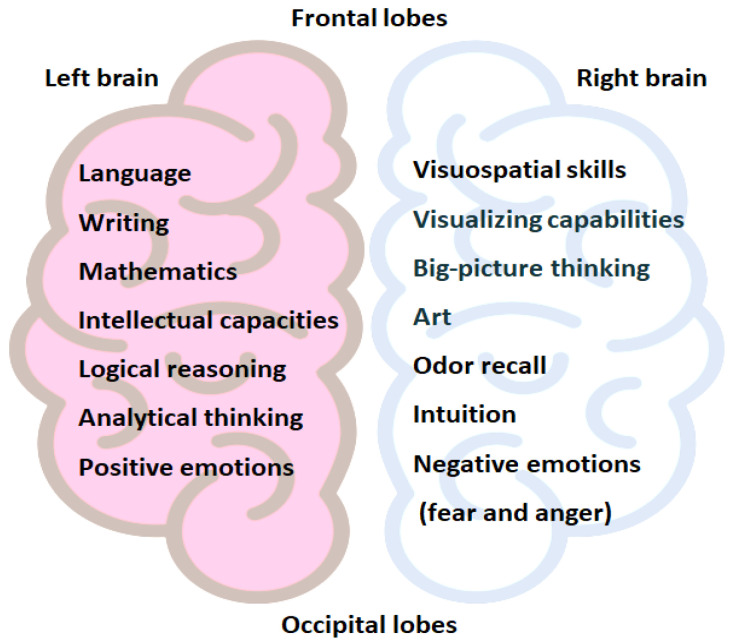
An illustration showcasing the dominance of brain-hemisphere capabilities in humans. Note that left or right brain dominance in specific skills implies the involvement of the contralateral hemisphere in that particular skill as well. Indeed, many of these skills may rely on bilateral brain activity.

Brain laterality is defined as the structural and functional asymmetry of the left–right brain hemispheres, a phenomenon that was observed not only in mammals, but also in birds, fish, and insects [5]. Anatomical asymmetry is not limited to the brain, as it is a common phenomenon observed in the bronchial branches of the lungs, kidney, breasts, and fingerprints, along with the rightward positioning of the liver and the leftward positioning of the heart, stomach, pancreas, and spleen, among others. Genetic, epigenetic, and environmental factors have been suggested as developmental determinants of the asymmetric body plan [5,6]. In humans, the brain laterality correlates to the left-brain dominance in language skills, writing, mathematics, intellectual capabilities, and probably with positive emotions, while the right brain may have dominance in art, visuospatial skills, likely negative emotions (such as fear and anger), and odor recall [7,8], among others, as shown in Figure 1. In fact, in real life, neuronal circuits are not required to be the same in each hemisphere, particularly in the frontal lobe, which is not involved in simple localized functions such as sensing and movement. In this way, each hemisphere can have its own additional specialized circuits and functions to enhance the brain functionality regarding attention and cognition. For example, the right brain can screen and hold vigilance and attention to dangers in the surrounding environment, while the left brain is engaged with talking, thinking, and planning at the same time.

Where brain hemisphere asymmetry/laterality is a well-conserved biological feature of normal brain development, it starts long before birth: at 8 weeks post-conception [9]. In fact, ultrasound studies in this early embryonic stage revealed that right arm movements are more frequent in 85% of fetuses [9]. At 13 weeks post-conception 90% of fetuses predominantly use their right hand for thumb sucking, and 5.4% (12 out of 224) prefer to use their left thumb, and this trend remains stable in the following months of the embryonic period [10]. Follow-up studies uncovered that two-thirds of those fetuses that used their left thumb (10 out of 15) become left-handed at school age, and most of those who used their right thumb remained right-handed [11]. Given that multiple lines of evidence indicate the disruption of the normal pattern of brain asymmetry in several mental illnesses, this comprehensive review addresses these alterations and delves into the potential mechanisms involved in brain-laterality formation. Furthermore, it addresses therapeutic interventions that may help minimize the functional impacts of brain-laterality disruption in specific mental illnesses.

## 2. Methods

Our primary focus here is to summarize the brain laterality disruption observed in SCZ, BD, ADHD, and OCD, as multiple studies, including meta-analyses, have addressed this condition. To gather the relevant information for this comprehensive review, we conducted separate searches in the PubMed database using the terms “laterality” or “asymmetry” along with MRI and the name of the specific disorder. After excluding review papers, case reports, and animal studies, over 700 studies were identified for further assessment, with 440 related to SCZ, 100 for each BD and ADHD, and 55 for OCD, from 2001 up to December 2023. Following the review of the abstracts, relevant studies were further examined to obtain detailed information, much of which is presented in the tables (31 SCZ, and 12 ADHD) or text (13 BD, and 4 OCD) of this review. Note that a few historically important studies predating the year 2001 were also included in this work.

In the context of epigenetic modifications as the underlying mechanisms for the formation of brain laterality, we conducted a systematic review using the terms “epigenetic”, “DNA methylation”, “miRNA”, and “histone” separately, along with the term “brain laterality” or “asymmetry” and found 125 studies. Among them, we identified 12 studies relevant to this article after excluding a large number of reviews and studies related to lateralized brain cancer or eye diseases. By employing this approach with the inclusion of specific brain disorder and illness names (schizophrenia, bipolar disorder, ADHD, and OCD or obsessive), we identified almost 100 studies discussing various epigenetic modifications in relation to laterality or asymmetry. Approximately half of these studies were related to SCZ (49 SCZ, 24 BD, 17 ADHD, and 7 OCD). After excluding reviews and animal studies, we found 14 relevant human studies that provided applicable information for inclusion in this article. We also identified 28 studies related to autism (mostly reviews or animal studies) that were not among the main theme of this study but contained applicable information. The same approach (utilizing the search terms epigenetic, histone, DNA methylation, or miRNA and rTMS or ECT) was also employed to identify the epigenetic effects of interventions discussed in Section 7, encompassing a total of 22 relevant studies out of 123, including both animal and human research findings.

## 3. Evidence Linking the Loss of Brain Asymmetry to Mental Illnesses

### 3.1. Schizophrenia (SCZ)

SCZ is one of the most prominent mental illnesses exhibiting distinct brain laterality alterations. Interestingly, studies on “fluctuating asymmetry” which represents the degree to which the right and left side of the body are asymmetric (a sign of developmental instability) uncovered that aberrant asymmetry is not brain-specific in SCZ. Indeed, it has been shown that anatomical asymmetries in fingers and ears in SCZ patients are more common than in control subjects, and these aberrations are associated with more hallucination and thought-disorganization scores [12]. Additionally, SCZ patients exhibit more fluctuating asymmetry and atypical hand skills compared to the control subjects, and meta-analyses revealed that left handedness and non-right-footedness are more common in individuals with SCZ [13].

Along with these clinical observations, the development of advanced imaging and functional studies in the last two decades (in particular functional magnetic resonance imaging (fMRI) and scalp electrical-activity mapping) were instrumental in finding the asymmetric brain structural counterparts of psychiatric illnesses. In SCZ, the most serious psychiatric illness with well-established left–right loss of the typical pattern of functional brain asymmetry (Table 1), it was uncovered that, in particular, the left brain hemisphere is affected in disease states (e.g., atrophy of dorsolateral frontal cortex and cingulate gyrus) using different imaging techniques [14,15,16,17,18]. A recent large-scale analysis of 35 studies of MRI data from 5080 SCZ cases vs. 6015 controls also revealed thinner left-hemispheric cortices in SCZ versus controls [19]. The results of three decades of research on this topic are summarized in Table 1, highlighting the anatomical and functional disruption of brain laterality in SCZ.

### 3.2. Bipolar Disorder (BD)

A recent large study of 2174 BD patients reported that non-right handedness is associated with an earlier age of illness onset and chronic substance-use disorder [48]. A recent MRI study also uncovered that while both SCZ and BD patients exhibit aberrant brain asymmetry, the pattern of the asymmetry in BD patients is different from that of SCZ; there was a higher asymmetry index for the cerebellum in patients with SCZ vs. BD, but a higher asymmetry index for the anterior cingulate cortex and Brodmann areas 6, 11, and 37 in patients with BD versus patients with SCZ [49]. Additionally, a study of auditory evoked fields in BD reported a reduced left–right hemisphere asymmetry of the primary auditory cortex, but not of the secondary auditory cortex, which is affected in SCZ [50]. A study analyzing the fMRI data of BD patients found that there were greater alterations in the “topological properties of the right” vesus the left hemisphere when compared to the control subjects [51]. Another fMRI study also reported more disturbances in the right hemisphere in BD [52]. In a line-bisection task using the right hand, in contrast to SCZ patients who had rightward deviation, BD patients exhibited more leftward deviation vesus controls [53], indicating an abnormal function of the right brain hemisphere in BD. 

Analysis of the cerebral blood flow of gray matter also revealed that an increase in right fronto-limbic cerebral blood flow affects working-memory functions in BD [54]. These observations are in line with the conclusion of a copmprehensive review confirming that mania is linked to right-hemisphere lesions [55]. Meanwhile, MRI studies in BD showed that the left amygdala volume was larger, but left anterior cingulate volume was smaller compared to the control subjects [56,57]. Another MRI study reported that the left hypothalamus is also larger in BD as well as in unmedicated MDD patients compared to controls [58]. These inconclusive findings might be due to the diverse nature of brain dysfunction in the manic versus depressed phases in BD patients. In a similar line of observation, a recent review of 10 resting-state EEG along with task-related fMRI studies in BD concluded the cerebral dominance of “the left dorsolateral prefrontal cortex and dorsal anterior cingulate cortex” in the manic phase, but cerebral dominance of the “right dorsolateral prefrontal cortex, orbitofrontal cortex and temporal pole” in the depressive phase of BD [59]. An earlier longitudinal qEEG study of BD also reported that the asymmetry of the frontotemporal slow-wave activity in depression exhibits an opposite direction compared to mania [60].

### 3.3. Attention Deficit/Hyperactivity Disorder (ADHD)

Similar to SCZ and BD, a recent meta-analysis has shown that non-right-handedness is more common in individuals with ADHD versus the control subjects [61]. A recent meta-analyis of seven MRI studies of 324 patients with ADHD verus 303 control subjects also provided evidence for brain laterality alterations in ADHD [62]. Another meta-analysis of 39 studies, involving 1933 cases of ADHD and 1829 control subjects, found that children diagnosed with ADHD demonstrated reduced rightward asymmetries of the surface areas of medial orbitofrontal cortex and the total hemispheric surface [63]. Several other studies have also identified atypical brain asymmetry in individuals with ADHD using various methods, as outlined in Table 2.

### 3.4. Obsessive Compulsive Disorder (OCD) 

In patients with OCD, one of the earliest MRI studies reported the lack of normal brain hemispheric asymmetry of the hippocampus–amygdala complex in 26 cases vs. 26 controls [76]. Subdsequent studies involving 72 OCD cases vs. 72 control subjects reported a reduced gray-matter volume of the left insulo-opercular region and a reduced volume of the amygdala in the right hemisphere in OCD with more aggressive obsessions and checking compulsions [77]. A meta-analysis of 28 fMRI studies during executive task performance in adult OCD vs. control subjects uncovered an increased activation of the left middle frontal gyrus and functional deficit of the right caudate body in OCD [78]. More recently, a large-scale analysis of 16 pediatric datasets, which included 501 children with OCD and 439 control subjects, reported more leftward volume asymmetry of the thalamus and less leftward asymmetry of the pallidum in those affected [79].

In summary, these lines of evidence confirmed by the meta-analysis of different studies support the disruption of brain laterality in major mental illnesses, including SCZ, BD, ADHD, OCD, and autism [47,62,80,81]. Furthermore, as abnormal brain lateralization in the planum temporale (a critical structure in auditory language processing) has been reported in patients with SCZ, it has been considered as a major cause for the onset of auditory verbal hallucinations [40,82]. Interestingly, peripheral counterparts of abnormal brain laterality in SCZ have been shown in several tissues. For example, approximately 95% of the general population has a clockwise hair whorl rotation. However, in patients with SCZ, this occurs randomly [83,84,85]. An aberrant feature of fingerprints and fluctuation asymmetry, along with other minor physical anomalies, has also been reported in SCZ, ASD, and ADHD [86,87,88,89,90,91]. Therefore, it appears that the disruption of laterality establishment is not brain-specific in these neurodevelopmental illnesses.

## 4. Consequences and Origin of Brain Laterality Disruption in Mental Illnesses

The emerging lines of evidence suggest that brain laterality aberrations may be associated with various mental illnesses. Some neuroscientists suggested that a disruption in the logical dominance of the left brain in right-handed individuals (and likely the reverse is true in left handed individuals) give rise to lack of insight related to “wishes” and “fears”, which may lead to delusional thinking (e.g., grandiosity and persecutory delusions, respectively) while perceptual distortions may be perceived as hallucinations in psychosis [92]. In this way, “an individual’s own thoughts [which may come from the non-dominant brain hemisphere and allied with whishes or fears] are perceived as an external intruding voice” [30] in SCZ or other psychotic illnesses. In non-psychotic illnesses such as OCD, it is conceivable that intrusive thoughts, such as the fear of contamination or uncertainty, may arise from the non-dominant brain hemisphere, with an inability of the dominant hemisphere to suppress them. In ADHD, as the left brain is busy with thinking or reading, an external noise or image cannot be filtered out by the dominant hemisphere. Thus, it is reasonable to propose that, as the left brain is engaged in tasks such as thinking or reading, this dominant hemisphere may struggle to filter out external stimuli such as noises or eyesight and images, which naturally are displayed to the right hemisphere.

With respect to potential causes of brain laterality disruption in mental illnesses, until the last decade, mutations of genes responsible for the establishment of brain laterality was the main hypothesis to describe its origin, in particular in SCZ [92]. However, numerous genetic association and linkage studies, along with more recent very large genome-wide genetic association studies (GWAS) have failed to pinpoint any gene with a reasonable effect size (even 1%) attributed to SCZ or other major mental illnesses [93,94]. Although in a recent GWAS, 48 genetic variants were associated with handedness and there were suggestive correlations between left-handedness and neuropsychiatric traits, again all of them had very small effect sizes, <0.5% [95], suggesting that each of the genetic variations are trivial risk factors superimposed on the impact of other major player(s). Large-scale twin studies involving more than ten thousand cases estimated that the heritability of handedness could be around 25% [96,97]. On the other hand, environmental factors such as rearing conditions, life experiences, and even the methamphetamine challenge (known to induce psychosis) can influence the development of cortical laterality of the brain. This, in turn, can lead to disturbances in the postnatal maturation of lateralized brain functions and, consequently, certain psychopathological behaviors [98]. The involvement of environmental factors raises the possibility of the contribution of epigenetic regulation to the establishment and/or maintenance of the left/right body plan and the disruption of brain laterality in major mental illnesses.

## 5. Epigenetic Regulation of the Establishment and/or Maintenance of Brain Laterality

Epigenetic modifications are key contributors to cell differentiation for the establishment and maintenance of cells’ structural/functional diversity in multicellular organisms. This could be true for the epigenetic modifications of genes related to the structural/functional asymmetry of brain hemispheres, which are affected in SCZ and BD [99,100,101]. Like the genetic information, epigenetic information (called epigenetic memory) is also heritable [102,103,104,105]. Nonetheless, they are influenced by diverse environmental factors such as malnutrition, infections, chemical contaminations, ecological conditions, and likely the social environment, as reviewed elsewhere [99,100,101,106,107]. For instance, a deficiency in methionine, choline, folic acid, and vitamin B12, which are essential for the functioning of the methylation machinery, as well as inflammation and oxidative stress induced by a pathological gut microbiome, can lead to epigenetic changes and the loss of epigenetic memory [99,100,101,108,109,110].

Before the emergence of evidence supporting the potential roles of epigenetic alterations in the pathogenesis of neuropsychiatric illnesses, it has been proposed that specific genetic variations are associated with structural brain-laterality alterations (e.g., the Xq21.3/Yp duplicative transposition of the sex chromosomes in SCZ) [111,112]. There has also been evidence for genetic susceptibility to aberrant language asymmetry patterns in parents of children with autism spectrum disorder [113]. Nonetheless, a study in *C. elegans* has uncovered that miRNAs are also involved in the specification of left–right neuronal function, which begins very early, even at the 4-cell stage following the determination of the anterior–posterior axis at the 2-cell stage [114]. More recent experimental evidence revealed that as brain laterality is affected by environmental factors such as multigenerational stress in rats, epigenetic regulations may be involved in the establishment or disruption of brain laterality [115]. In male gerbils, it was shown that the establishment of asymmetric serotonin fiber density in the left versus right brain was similarly compromised both by early life methamphetamine use and restricted environmental and social-rearing conditions, which has been proposed to be epigenetic in origin [98]. In mice, feeding ethanol in the first eight days of the gestational period also altered the expression of 23 genes and the DNA methylation of the promoter region of several affected genes along with the altered expression of three miRNAs. These alterations were accompanied by the enlargement of the left hippocampus but a smaller left olfactory bulb [116]. It is important to note that early life perturbations, such as monocular deprivation during visual-cortex development, can similarly alter the DNA methylation and hydroxymethylation of specific genes in mice, which could be partially prevented by DNMT (DNA methyltransferase) inhibition [117]. Monocular deprivation in mice leads to a decrease in miR-132 expression in the contralateral cortex, as well, which can be mitigated by the use of miR-132 mimic oligonucleotides, preserving ocular plasticity [118]. In addition, the early life plasticity of visual-cortex-mediated ocular dominance can be partially preserved by miRNA-29a inhibition, while its expression is increased by age and stabilizes the already formed visual-cortex circuits [119]. Nevertheless, histone H3-H4 acetylation and H3 phosphorylation are also involved in the plasticity of visual cortical circuits in mice [120].

In humans, a genome-wide mRNA expression and DNA methylation analyses of the “cervical and anterior thoracal spinal cord segments” of five fetuses uncovered a developmental-dependent asymmetric expression of collagen genes and the TGF-β-signaling pathway, which are regulated by asymmetric miRNA expression and the DNA methylation of CpG islands [7]. Another DNA-methylation-profiling study of the neurons of the human frontal cortex identified that the left hemisphere exhibits more methylation, and >2500 CpG sites exhibit asymmetric DNA methylation in the left vs. the right brain, and this asymmetry is diminished progressively by aging [121]. However, in Parkinson’s disease, this change is more than controls, particularly in the left brain, and the number of asymmetric CpGs is twice as high versus controls, including the CpGs of genes known to be involved in the disease pathogenesis. Furthermore, the differences were more frequent in the affected hemisphere of these patients associated with more gene-expression differences versus the control subjects [121].

Altogether, these data provide evidence that well-known mechanisms of epigenetic regulations, including DNA methylation, histone modifications, and miRNA interference, participate in the establishment and/or maintenance of brain laterality, which is disrupted in major mental illnesses, as described in the following section.

## 6. Interrupted Epigenetic Regulation of Brain Laterality in Mental Illnesses

Gene-specific promoter methylation analysis of DNA extracted from the saliva of healthy individuals for *KIAA0319*, a gene known to be involved in ciliogenesis (important in left–right body specification during the early embryonic stage) and affected in dyslexia, revealed that its sex-specific promoter CpG methylation is linked to the regulation of cognitive processes and dichotic listening during the “forced attention condition” [122]. This gene is located in chromosome 6 next to *TDP2* and *ACOT13* genes, and a risk haplotype of these three genes is associated with a 40% decrease in *KIAA0319* expression and thus dyslexia [122]. Additionally, a haplotype of the methyltransferase *SETDB2* (in particular, its homozygous A allele of the sequence variant rs4942830) which is involved in histone H3 methylation (an epigenetic mark) is linked to unusual handedness in humans [123].

More evidence in support of the epigenetic regulation/determination of language and handedness comes from a meta-analysis of the whole-blood DNA methylation of almost 4000 adults, reporting that the DNA methylation of CpG sites closed to the genetic variants associated with handedness were significantly linked to left-handedness compared to other CpG sites [124]. A more recent genome-wide DNA methylation analysis of neonate’s cord blood revealed that the methylation of *KCTD*, *SDCCAG8*, and *GLRX* genes which are involved in brain functions are prospectively associated with the left ventricle volume at age 10 years [125], with an enlargement in SCZ (see Table 1).

In gene-specific epigenetic analyses, the DNA methylation of the putative promoter region of *LRRTM1* (leucine-rich repeat transmembrane 1), an imprinted gene expressed in the brain and linked to SCZ risk and handedness in dyslexia, was associated with mixed handedness in nonclinical samples [126]. The DNA hypomethylation of the *LRRTM1* promoter region in blood cells has been linked to SCZ as well [127]. Moreover, the DNA methylation of the promoter of *AHI1* (a gene related to cilia functions contributing to brain laterality formation) in buccal cells could predict language lateralization association with schizotypy in humans [6]. The promoter DNA methylation of *DBH* (dopamine beta hydroxylase gene) in buccal cells has also been linked to line-bisection deviations in healthy individuals during right-aligned trials. Note that, in this task (during which individuals are asked to detect the center of 10 or more horizontal lines on a paper), healthy individuals exhibit leftward attentional bias, as the right brain hemisphere is dominant for visuospatial attention [128]. However, patients with ADHD exhibit rightward attentional bias [129].

In addition to these lines of evidence, lateralized epigenetic regulations of several other genes were found to be disrupted in the brains of patients with major psychiatric disorders. For example, the loss of brain asymmetry in *TGFB2* expression due to its altered promoter DNA methylation along with the disruption of the lateralized expression of many collagen genes has been linked to SCZ and BD pathogenesis [130]. As mentioned before [7], collagen and TGFβ-signaling genes are key to the establishment of the left–right asymmetry of the cervical and anterior thoracic spinal cords in early embryonic periods, as well.

In other studies, there are reports on the loss of the lateralized expression of *DTNBP1* due to its altered promoter DNA methylation in SCZ and BD patients compared to the control subjects [99], in addition to the loss of the lateralized expression of (i) *MB-COMT* (membrane-bound COMT), (ii) *HTR2A* (serotonin type-2 receptor, reduced in the left brain of SCZ and right brain of BD patients), and (iii) *SLC6A4* (*5-HTT*, serotonin transporter) in the post-mortem brains of patients with SCZ and BD, all associated with corresponding DNA methylation changes versus the control subjects [131,132,133].

Altogether, these data suggest that epigenetic modulation participates in brain asymmetric configuration, and the loss of lateralized epigenetic regulations contribute to illness pathogenesis in major mental illnesses. Therefore, while pharmacological therapy affects both brain hemispheres, and many psychiatric symptoms remain untreated with current psychiatric drugs, it is necessary to consider other therapeutic options with unilateral applications in the treatment of specific mental illnesses.

## 7. Potential Therapeutic Approaches with Unilateral Applications and Epigenetic Modulation

Among the available therapeutic options that can be applied unilaterally or asymmetrically in a patient-specific manner, Electroconvulsive Therapy (ECT) and repetitive transcranial magnetic stimulation (rTMS) are currently utilized in clinical settings. The history of the application of ECT in the treatment of mood disorders and SCZ dates back almost 80 years [134,135]. During this treatment, a 100-volt electric current is applied to the frontal lobes for 1–3 s following a brief anesthesia accompanied with oxygenation and the use of a muscle relaxant (e.g., succinylcholine), resulting in a grand mal seizure that is essential for the therapeutic effects of ECT. Several sessions of this treatment are administered over a period of 2–3 weeks, typically every other day. Many institutions utilize unilateral ECT (to the right hemisphere) to reduce its temporary impact on recent memory. Research has demonstrated that unilateral ECT is as effective as bilateral ECT [136]. In rTMS, specific brain regions, based on the nature of the illness, are exposed to a magnetic field that stimulates neuronal activation. The targeted pathways can be inhibitory or stimulatory, and unlike ECT, rTMS has localized activity rather than generalized. The effectiveness of rTMS has been demonstrated in several neuropsychiatric illnesses such as SCZ, depression, BD, and Parkinson’s disease, among others [137,138,139].

There is adequate experimental evidence indicating that these therapeutics can modify epigenetic and/or gene expression status. For example, the exposure of human neuronal cells to a low-frequency pulsed magnetic field could decrease the DNA methylation of LINE-1 (long interspersed nuclear element-1), which was more significant if combined with oxidative stress [140]. The DNA methylation of LINE-1 is a known indicator of a healthier lifestyle and the global DNA methylation of the genome [141]. As rTMS is an effective therapy in multiple neuropsychiatric illnesses, including Alzheimer’s disease (AD), an animal study uncovered that rTMS can improve neuronal activity by increasing cFos expression, decrease astrocyte and microglia activation, and reduce Aβ deposits in high-fat diet-induced mice models of AD [142]. Additionally, in chronic unpredictable stress-induced depression in mice, rTMS could improve depression and dendritic remodeling associated with an increase in histone H3 trimethylation (H3K9me3, a repressor epigenetic mark) in the frontal cortex along with DNA methylation changes in the hippocampus, both globally and in mature neurons [143]. In humans, rTMS therapy could alter the DNA methylation of the COMT gene in the blood cells of patients with PTSD, where reduced DNA methylation over time was associated with a better response [144].

Regarding other types of epigenetic alterations, 1000 stimuli over 3 days with rTMS in vitro on the neural progenitor cells of the rat hippocampus could induce cell proliferation mediated by miR-106b upregulation [145]. In vivo studies revealed that both single sessions of 20 min and repetitive sessions (five days) of rTMS to the left hemisphere of rats could alter the expression of several dozen mRNAs (related to brain functions) that could be attributed to the expression changes of a dozen miRNAs in the cerebral cortex compared to sham rTMS [146]. In scopolamine-induced cognitive dysfunction in an AD model mice, it was shown that rTMS mitigated tissue damage, mediated by miR-567 downregulation, and restoring *BDNF* and *NGF* production [147]. In 6-OHDA-induced Parkinson’s disease in mice, rTMS could alter the expression of many miRNAs, among which in particular miR-409-3p suppression was associated with attenuated brain injuries, increased neuron numbers, and an improvement in cognitive functions [148]. Treatment with rTMS could increase miR-25 expression, enhancing adult neural stem cell proliferation in the rat’s subventricular zone after focal cerebral ischemia, as well [149]. Additionally, rTMS could mitigate stroke-induced cognitive decline in rats, mediated by a decrease in miR-409-3p level in the affected brain region. However, rTMS could also alter the expression of >350 other miRNAs (104 upregulated and 249 downregulated) [150]. In another study, rTMS mitigated brain ischemic stroke injury in rats by preventing M1 microglia polarization mediated by “let-7b-5p/HMGA2/NF-κB signaling” regulation [151]. In a recent study in mice imposed by status epilepticus, low frequency rTMS was found to alter the expression levels of 510 mRNAs, 1615 lncRNAs, and 17 miRNAs, including those linked to GABA-A receptor activity and immune functions [152].

In humans, a randomized and double-blind study reported that the expression of miRNA-let-7d which was higher in the blood cells of patients with ADHD was reduced after six weeks of rTMS or atomoxetine treatment [153]. In medication-resistant patients with depression undergoing rTMS therapy, the expression changes of miR-146a-5p (an anti-inflammatory miRNA) as well as miR-16-5p and miR-93-5p were associated with a better response to rTMS [154].

Regarding the epigenetic effects of ECT, various mechanisms of epigenetic regulations, including miRNAs, histone modifications, and the DNA methylation of several genes were correlated with ECT therapeutic effects in multiple/different animal studies, as reviewed a decade ago by de Jong et. [155]. In a more recent human study using a whole-genome DNA methylation analysis, the altered DNA methylation of ten genes was attributed to ECT response in MDD, including three genes encoding long non-coding RNAs [156]. In another study, DNA methylation changes of *PLAT, SERPINE1*, and *CREB* in specific blood immune cells (e.g., natural killer cells and T cells, but not whole-blood cells) were associated with the ECT response in drug-resistant patients with MDD [157]. A recent review of nine epigenetic studies related to ECT effects in the peripheral samples of patients with MDD also found that the DNA methylation of five genes (*BDNF*, *FKBP5*, *RNF213M*, *S100A10*, and *TNKS*), and the alteration of three miRNAs (miR-24, miR-106a, and miR-126) are associated with ECT response [158].

It is also important to note that there is some evidence that antipsychotic drugs may also be involved in the readjustment of some aspects of the disrupted brain laterality in psychiatric illness. For example, it has been shown that antipsychotic drugs could decrease right hemi-spatial inattention in right-handed SCZ patients in visuospatial tasks [159]. Another report, based on a meta-analysis of nearly 800 subjects, concluded that the volume of the left globus pallidus is directly associated with the use of antipsychotic drugs in SCZ patients, while the volume of the right hippocampus is inversely associated with it [160]. A more recent study also reported that six weeks of aripiprazole treatment (an atypical antipsychotic drug) could increase the lateralization index of the inferior frontal gyrus in a semantic task in SCZ (associated with an improvement in verbal fluency) as the result of a decrease in the activation of the right inferior frontal gyrus [161]. Although these studies have not analyzed the epigenetic counterpart of these lateralized alterations in response to antipsychotic drugs, some other studies demonstrated that these drugs can modify epigenetic alterations linked to asymmetric brains in SCZ and BD. For example, as there is an inverse correlation between the DNA methylation of *5-HTT*-linked polymorphic region and the left-amygdala volume (i.e., its DNA methylation reduces left-amygdala volume) [162], antipsychotic drug use is associated with less methylation of the *5-HTT* (*SLC6A4*) promoter region both in postmortem brains and the saliva of patients with SCZ and BD [133]. The promoter DNA methylation of *HTR2A* in the postmortem brains of SCZ patients, which reduces its expression, is also decreased by antipsychotic drugs where *HTR2A* exhibits lateralized expression in SCZ and BD (reduced in left and right brains, in SCZ and BD, respectively, compared to the control subjects [132].

In addition to ECT and rTMS, there are other therapeutic approaches that can be used unilaterally for the treatment of patients with asymmetric brain dysfunctions. For example, neurofeedback is an effective therapeutic approach for conditions such as ADHD, depression (including MDD), and SCZ [163,164,165]. Recent meta-analyses have concluded that physical exercise is also effective in improving cognitive functions in conditions such as ADHD, depression (including MDD), schizophrenia (SCZ), and other mental disorders [166,167,168,169]. Hence, these therapeutic approaches can be tailored more specifically for unilateral applications as complementary strategies in the treatment of patients with asymmetric brain dysfunctions. While epigenetic modifications are well-known mechanisms of the action of physical exercise in brain functions [170,171], the potential epigenetic mechanisms of neurofeedback have not yet been clearly elucidated. Altogether, these studies address the potential application of different therapeutics that could be employed unilaterally in psychiatric patients with the loss or reversal of brain asymmetry involving epigenetic mechanisms. Nevertheless, there are several challenges that will be discussed in the following section.

## 8. Challenges in Applying Brain Laterality Research to Clinical Practice

There are several challenges that may impede the clinical application of research findings related to brain asymmetry for enhancing mental health. While advances in genetic strategies have hastened the identification of molecular asymmetry in the brains of patients with various mental disorders, large-scale approaches for assessing the developmental basis of brain laterality establishment remain a challenge. Recognizing left-right differences in gene expression during brain development is difficult due to their transient nature and localization to specific areas of the brain [172]. While current data on the epigenetic origins of brain laterality in humans is limited to fetuses at 10–14 weeks gestational age, novel single-cell analysis techniques may have the potential to trace its establishment back even earlier. For a more in-depth analysis, it is crucial to note that during the first mitotic event, cell division is asymmetric due to the asymmetric distribution of the centromere, more specifically, a centromere-specific histone-3 variant as an epigenetic regulatory factor [173]. Therefore, akin to *C. elegans*, the origin of left–right specification could be traced back to the first or second (after anterior–posterior specification at the first division) mitotic cell division. Despite practical obstacles in identifying potential laterality disruption in such early developmental periods in human fetuses, new methods for generating brain organoids with clear left–right and anterior–posterior specification from the iPSC lines of humans [174] with diverse neurodevelopmental illnesses may be instrumental in tracing the origin of laterality disruption.

Another challenge is that research in one species occurs in isolated settings and may differ from findings in other animal species or humans. Therefore, there is a need to bridge the gap between laterality research in various species, including small animals, non-human primate models, and human subjects. A potential solution is a comparative analysis of laterality formation across a variety of species, which may result in moderate to significant scientific advancements. Additional information about laterality and asymmetries in other species can assist research scientists in exploring the evolutionary trajectory of this phenomenon [175]. Furthermore, there is still controversy and debate as to whether changes in asymmetries contribute to the symptoms of the illnesses or are an epiphenomenon of altered neurobiological events involved in their pathologies [176]. Additionally, a range of factors that affect both neuropsychiatric illnesses and brain-lateralization patterns are still a topic of debate. One hypothesis is to derive support from the shared genetic foundation for neuropsychiatric illnesses and atypical brain lateralization. In support of this notion, a connection among brain development, left-handedness, and structural connectivity patterns related to the pathogenesis of neuropsychiatric disorders with the rs199512 locus has been reported in a genome-wide association study [177]. However, it is also known that environmental factors participate in the development of neuropsychiatric illnesses and atypical lateralization. For example, Berretz et al. reported that early life and chronic stresses not only play a significant role in increasing the risk of neuropsychiatric illnesses, but are also among the leading causes of the altered functional and structural hemispheric asymmetries in these disorders [178]. As mentioned earlier, multigenerational psychological stress can also alter brain laterality through epigenetic modifications [120]. Therefore, future research should focus on the causal relationship between the genetic and epigenetic underpinnings of neuropsychiatric illnesses and alterations in brain laterality, while also considering a variety of environmental factors related interactions with the genome and the epigenome, affecting the normal pattern of brain asymmetry. Furthermore, by integrating information from cell-specific and large-scale databanks (such as brain hemispheric and cell-specific expression and epigenetic data) and conducting meta-analytical studies, neuroscientists may attain unbiased conclusions about the precise effects of external factors on the establishment or disruption of brain laterality. It is important to note that in this study, we have not assessed the risk of bias for the included studies. Therefore, this remains one of the important limitations of this review.

With respect to therapeutic interventions, while rTMS can be considered an effective therapy for multiple neuropsychiatric illnesses by exerting changes in epigenetic status and gene expression, it primarily targets superficial structures and has limited influence on deeper brain structures such as the insula and the anterior cingulate cortex [179]. Hence, concerns may arise regarding whether the use of current rTMS protocols is a valid approach to restore lateralized epigenetic alterations in deeper brain structures, as most major mental illnesses exhibit a distributed pathology throughout different brain areas and circuits, rather than being confined to a single brain area or specific sets of synapses and pathways [180]. The long-term safety, efficacy, as well as sustained epigenetic and gene-expression changes, and brain-function modulation after the discontinuation of therapy with this technique, are also unclear due to the lack of long-term follow-up studies [181]. It is also important to note that current rTMS treatment protocols are not well-designed to target lateralized brain functional alterations in mental illnesses. Therefore, further studies are needed to explore modified protocols with improved therapeutic effects for patients with lateralized brain dysfunctions.

In relation to ECT, while epigenetic modifications are among the promising mechanisms of this type of treatment, there are a large number of ECT-induced neuroanatomical changes. This makes it difficult to identify which changes are associated with ECT’s therapeutic effects versus those that are secondary or potentially the origin of its adverse effects [182]. Therefore, it is essential to precisely explore the mechanism of action of unilateral ECT during the treatment of mental disorders with an aberrant brain-lateralization pattern. While experimental evidence indicates that the efficacy of rTMS is less than ECT for the treatment of patients with mood disorders or psychosis [183,184], it should be noted that rTMS is less invasive, more cost-effective, and associated with fewer adverse events on cognitive functions compared to ECT [185,186]. Finally, it is also important to note that as the potential effects of asymmetric physical exercise or neurofeedback on mental illnesses are still being identified, the current techniques (including ECT and rTMS) may primarily serve to minimize functional laterality dysregulations rather than their anatomical or structural counterparts, especially if structural alterations are formed in the early stages of neurodevelopment.

## 9. Conclusions

In summary, structural and functional asymmetry, or laterality, in the brain is a widespread phenomenon observed in mammals, birds, fish, and insects. The development of the asymmetric brain involves genetic, epigenetic, and environmental factors. In practical terms, neuronal circuits are not necessarily duplicated in the other brain hemisphere, especially in the frontal lobes, which play limited roles in localized functions such as sensation and movement. Accordingly, each hemisphere may possess its own specialized circuits and functions, thereby enhancing the overall brain capacity. In humans, the lateralized functions of the brain begin as early as the 13th week post-conception. In adults, the left brain is dominant for linguistic skills, writing, mathematics, intelligence, and positive emotions, while the right brain is dominant in visuospatial skills, art, negative emotions, and odor recall. Moreover, when the left brain is occupied with tasks such as talking, thinking, and planning, the right brain can maintain vigilance and attention to potential dangers in the surrounding environment, which is crucial from an evolutionary perspective.

Multiple lines of evidence suggest that epigenetic mechanisms play a role in the establishment of brain laterality, and if disrupted, it could have detrimental impacts on brain functions. Overwhelming evidence also indicates that the normal patterns of brain anatomical and functional asymmetries are disrupted in specific mental illnesses such as SCZ, BD, ADHD, and OCD, which could be due to epigenetic aberrations. *TGFB2*, several collagen genes, *MB-COMT, HTR2A, DTNBP1*, and *5-HTT* are among the affected genes in the postmortem brains of patients with SCZ and/or BD. There is also a large amount of data supporting the idea that, beside pharmacological psychiatric drugs, physical intervention methods such as rTMS or ECT and even neurofeedback and physical exercise are capable of modifying epigenetic alterations associated with brain dysfunction. Hence, it is possible to use unilateral brain rTMS and ECT or other interventions to restore lateralized epigenetic alterations observed in mental illnesses. However, rTMS may offer greater therapeutic potential due to its ability to provide the site-specific stimulation of various brain regions with diverse magnetic intensities, offering more flexibility compared to ECT.

## Figures and Tables

**Table 1:** Supporting evidence for the loss of brain laterality in schizophrenia (SCZ).

Methods/Sample/(Author-Year)	Objective	Findings	Ref.
Postmortem brains 40/30/(Crow et al. 1989)	Ventricular size	Enlargement of left ventricle	[20]
Auditory evoked magnetic field/(Rockstroh et al. 1998)	Left-hemisphere activation to right-ear stimulation	Absence of contralateral dominance in response to auditory stimulation in SCZ	[21]
MRI, 16 drug-naïve SCZ/25 Cont./(Gilbert et al. 2001)	Thalamic volumes	Smaller thalamic volume in left central medial subdivision	[22]
fMRI, high-resolution 3D, SCZ/(Yotsu tsuji et al. 2003)	Ventricular size	Enlargement of left ventricle (male)	[23]
fMRI, 15 acutely ill SCZ/(Walter et al. 2003)	Cortical activation in verbal/spatial working-memory task	Absence of prefrontal lateralization in comparing verbal and spatial working memory	[24]
fMRI, high-functioning SCZ vs. Cont./(Weiss et al. 2004)	Cortical activation during verbal fluency	Left Broca in Cont., bilateral in SCZ	[25]
MRI 27 drug-naïve SCZ/(Prasad et al. 2005)	Functional outcome	Left DLPFC volume predict functions in SCZ	[26]
Postmortem DLFC of SCZ, 10/10/(Cullen et al. 2006)	Asymmetry of pyramidal cell density	Greater density in left side in Cont., less density or its reversal in SCZ (layer 3)	[27]
fMRI 24 SCZ/(Sommer et al. 2008)	Cerebral activation during auditory hallucination	Activation of right homologous of Broca’s but not Broca	[28]
Gamma synchrony in SCZ and 2.5 years later/(Williams et al. 2009)	Neuronal synchrony in first-episode SCZ	Progressive gray-matter loss in SCZ > in left, and progressive disruption of the laterality of early gamma synchrony	[29]
Mapping of scalp electrical activity in SCZ/(Angrilli et al. 2009)	Mean response time in linguistic tasks	Increased in SCZ, loss of left frontal activity during phonological tasks	[30]
fMRI, 35 first-episode drug-naïve SCZ/43 Cont./(van Veelen et al. 2011)	Measures of language lateralization	Reduced laterality index in SCZ, mostly in Broca’s and Wernicke’s areas (i.e., left)	[31]
(fMRI) during an auditory verb-generation/task (Bleich-Cohen et al. 2012)	Language-related activation in BA44/45 in inferior frontal gyrus	Reduced language asymmetry in SCZ	[32]
fMRI with speech–listening paradigm, 27/54/(Alary et al. 2013)	Functional laterality indices calculation	Decreased leftward hemispheric lateralization in SCZ	[33]
fMRI with a speech–listening paradigm in 31 SCZ vs. Cont. (Royer et al. 2015)	Functional laterality and gray-matter volume asymmetry indices	Decreased leftward functional hemispheric lateralization in SCZ	[34]
T1-weighted images, 884 SCZ/1680 Cont./(Okada et al. 2016)	Subcortical volumetric differences in SCZ vs. controls	SCZ-specific leftward asymmetry for pallidum vs. controls	[35]
High-resolution T1 structural images. 24 SCZ/26 Cont./(Núñez et al. 2017)	Global gray- and white-matter asymmetry	More global gray-matter asymmetry in SCZ vs. controls	[36]
Near-infrared spectroscopy, 28 first-episode SCZ/33 Cont./(Chou et al. 2017)	Laterality index during verbal fluency test	Reduced laterality index in SCZ vs. controls	[37]
Resting-state fMRI. 41 SCZ/35 Cont./(Son et al. 2017)	Examination of mean connectivity values	Reduced connectivity values of both left and right frontoparietal networks	[38]
Hemispheric white-matter topology. 116 SCZ/66 Cont./(Sun et al. 2017)	Estimate the hemispheric topological properties	Reduced hemispheric asymmetry of global integration in SCZ	[39]
Resting-state fMRI signal. 180 SCZ/358 Cont./(Xie et al. 2018)	Measuring lateralization index using standard deviations of resting-state fMRI activity	Abnormal variability and lateralization, “higher in left inferior temporal, left fusiform, and right superior medial frontal cortex”	[40]
Meta-analysis of T1-weighted structural brain scans, 1985 SCZ/(Walton et al. 2018)	Measuring medial orbitofrontal cortex thickness, meta-analysis of 17 studies	Left-side thickness is associated with negative symptoms and overall illness severity	[41]
Diffusion kurtosis imaging, 18 SCZ/19 Cont./(McKenna, et al. 2020)	Gray matter microstructural lateralization in male chronic SCZ	Reduced hemispherical asymmetry in SCZ	[42]
Diffusion tensor imaging, 50 SCZ/58 Cont./(Li et al. 2021)	Quantitative measure of hemispheric lateralization	Disrupted hemispheric asymmetry in SCZ in regions linked to emotion, memory, and visual processes	[43]
fMRI, 39 SCZ/42 Cont./(Potvin et al. 2021)	Functional connectivity study on visuospatial processing	Dysconnectivity between left and right sup. frontal gyrus (SFG) and left SFG and left sup. parietal lob.	[44]
3T MRI, 199/161/(Roeske et al. 2021)	Incomplete hippocampal inversion	Left brain, 27% in SCZ, but 15% in Cont.	[45]
Meta-analysis of 87 studies totaling 35,501 individuals/(Packheiser, et al. 2021)	Study of left–right or mixed handedness	No relation between handedness and depression	[46]
Meta-analysis of T1-weighted structural MRI scans, 2833 SCZ/3929 Cont./(Gutman et al. 2022)	Deep brain structural shape and asymmetry abnormalities	Exaggerated asymmetry across hippocampus, amygdala, and thalamus and diminished asymmetry in ventral striatum, ventral, and dorsal thalamus in SCZ	[47]
MRI, analysis of 35 studies, 5080 SCZ/6015 Cont./(Schijven et al. 2023)	Hemispheric cortex size and structure	Thinner left-hemispheric cortices in SCZ	[19]

**Table 2:** Abnormal brain asymmetry in patients with ADHD uncovered using different techniques.

Methods/Sample/(Author-Year)	Objective	Findings	Ref.
Structural brain MRI/341 ADHD vs. 508 Cont. and diffusion tensor imaging (DTI) in 104 subjects/(Douglas et al. 2018)	Volumetric measures of many regions on brain hemispheres	Differences in the asymmetry of cingulum, superior and inferior longitudinal fasciculus, and cortico-spinal tracts in DTI (improved by medication); more asymmetry in hippocampus, thalamus, caudate, and amygdala in morphometric analyses	[64]
Lateralized lexical decision task/77 ADHD/(Mohamed et al. 2016)	Visual-field advantage (better performance for the right vs. the left visual field)	Reduced right-visual-field advantage during slow-rate stimuli presentation in individuals with higher ADHD scores	[65]
MRI/119 adult ADHD vs. 107 Cont./(Onnink et al. 2014)	Gray- and white-matter volumes of different brain regions	Reduced right-caudate volume in male ADHD vs. controls	[66]
Dichotic listening task/19 unmedicated ADHD vs. 19 Cont./(Hale et al. 2006)	Measuring “hemispheric differences in word and emotion recognition” (hemispheric specialization)	Reduced left-hemisphere specialization; reduced left-hemisphere interference in linguistic stimuli (i.e., increased-right, but decreased-left hemisphere contribution)	[67]
Baseline EEG activity/117 unmedicated children with ADHD/(Baving et al. 1999)	Frontal-brain-activation analysis	Reduced right-lateralized frontal activation in boys but increased in girls	[68]
Rapid, event-related fMRI/16 drug-naïve adolescents with ADHD vs. 21 Cont./(Rubia et al. 2005)	Brain-activation status during inhibitory challenge	Reduced right inferior prefrontal-cortex activation during successful motor-response inhibition; reduced precuneus and posterior cingulate gyrus activation during inhibition failure	[69]
Post-error processing/30 students with higher vs. 26 with lower scores of the ADHD index/Mohamed et al. 2016)	Lateralized lexical decision task with a fast and slower stimulus-presentation rate	Left-hemisphere failure to compensate for errors (post-error response adjustments) in ADHD	[65]
EEG alpha asymmetry at rest and during the Conner’s Continuous Performance test/24 familial and 26 non-familial ADHD/(Hale et al. 2010)	Measuring brain laterality in familial vs. non-familial ADHD	Increased rightward frontal asymmetry in parent-affected ADHD and increased rightward parietal asymmetry in non-familial ADHD	[70]
fMRI during forward and backward digit spans/(Hale et al. 2007)	Complex executive operations via investigating repetition number	Increased activation of left linguistic-processing areas, right frontal and parietal cortices during forward digit span, but lack of activation of bilateral parietal regions during backward digit span	[71]
MRI, weighted hemispheric brain anatomical networks analysis/40 right-handed ADHD, 53 matched Cont./(Li et al. 2019)	Topology alterations in hemispheric white matter and its anatomical networks	Reduced hemispheric asymmetry of brain anatomical networks and lateralized hemispheric dysconnectivity	[72]
EEG beta asymmetry/35 adult ADHD vs. 104 Cont./(Hale et al. 2010)	EEG beta asymmetry during rest and active cognition in continuous performance task	Increased rightward beta asymmetry in inferior parietal regions (correlated with better performance); lack of association to left-biased processing in temporal-parietal region vs. Cont.	[73]
Meta-analysis of 27 whole-brain voxel-based morphometry or fMRI studies/931 ADHD, 822 Cont./(Norman et al. 2016)	Measuring structural and functional brain abnormalities	Reduced activation of the ventrolateral prefrontal cortex largely in the right brain hemisphere	[74]
fMRI/186 cases vs. 186 controls/(Li et al. 2023)	Patterns of working-memory-related functional brain activation	Reduced activation of the left inferior frontal gyrus	[75]

## Data Availability

Not applicable.
